# Mechanisms of manganese-induced neurotoxicity and the pursuit of neurotherapeutic strategies

**DOI:** 10.3389/fphar.2022.1011947

**Published:** 2022-12-20

**Authors:** Edward Pajarillo, Ivan Nyarko-Danquah, Alexis Digman, Harpreet Kaur Multani, Sanghoon Kim, Patric Gaspard, Michael Aschner, Eunsook Lee

**Affiliations:** ^1^ Department of Pharmaceutical Science, College of Pharmacy and Pharmaceutical Sciences, Florida A&M University, Tallahassee, FL, United States; ^2^ Department of Biology, College of Science and Technology, Florida A&M University, Tallahassee, FL, United States; ^3^ Department of Molecular Pharmacology, Albert Einstein College of Medicine, New York, NY, United States

**Keywords:** manganese, oxidative stress, inflammation, autophagy, mitophagy, excitotoxicity, apoptosis, neurotherapeutics

## Abstract

Chronic exposure to elevated levels of manganese *via* occupational or environmental settings causes a neurological disorder known as manganism, resembling the symptoms of Parkinson’s disease, such as motor deficits and cognitive impairment. Numerous studies have been conducted to characterize manganese’s neurotoxicity mechanisms in search of effective therapeutics, including natural and synthetic compounds to treat manganese toxicity. Several potential molecular targets of manganese toxicity at the epigenetic and transcriptional levels have been identified recently, which may contribute to develop more precise and effective gene therapies. This review updates findings on manganese-induced neurotoxicity mechanisms on intracellular insults such as oxidative stress, inflammation, excitotoxicity, and mitophagy, as well as transcriptional dysregulations involving Yin Yang 1, RE1-silencing transcription factor, transcription factor EB, and nuclear factor erythroid 2-related factor 2 that could be targets of manganese neurotoxicity therapies. This review also features intracellular proteins such as PTEN-inducible kinase 1, parkin, sirtuins, leucine-rich repeat kinase 2, and α-synuclein, which are associated with manganese-induced dysregulation of autophagy/mitophagy. In addition, newer therapeutic approaches to treat manganese’s neurotoxicity including natural and synthetic compounds modulating excitotoxicity, autophagy, and mitophagy, were reviewed. Taken together, in-depth mechanistic knowledge accompanied by advances in gene and drug delivery strategies will make significant progress in the development of reliable therapeutic interventions against manganese-induced neurotoxicity.

## 1 Introduction

Manganese (Mn) is an essential trace element in various cellular functions in the body, serving as a cofactor of several enzymes such as glutamine synthetase (Wedler and Denman, 1984) and Mn superoxide dismutase (MnSOD) (Buettner et al., 2006). The daily reference intake (2.3 mg/day for men and 1.8 mg/day for women) of Mn is required for physiological functions and acquired through a regular diet (Aschner and Aschner, 2005). However, chronic exposure to elevated level of Mn primarily *via* occupational and environmental settings (Lucchini et al., 2007; Lucchini et al., 2012) results in its accumulation in the basal ganglia of the brain, causing a neurological disorder referred to as manganism, resembling symptoms of Parkinson’s disease (PD) (Olanow, 2004; Aschner and Aschner, 2005; Bowman and Aschner, 2014). Chronic exposure to elevated level of Mn by inhalation in South African miners contributed to severity of parkinsonism-like symptoms, such as motor deficits (Racette et al., 2021a) as well as greater depression and anxiety (Racette et al., 2021b). Clinical studies have also shown that elevated level of Mn is correlated with cognitive and memory deficits in children and adults (Zhang et al., 2021; Heng et al., 2022), suggesting a link between Mn toxicity and neurocognitive disorders such as Alzheimer’s disease (AD) and dementia. The patients with a pathologically elevated level of Mn showed behavioral defects and irreversible neurological disorders (Olanow, 2004; Cersosimo and Koller, 2006; Kwakye et al., 2015; Harischandra et al., 2019a).

Mechanisms of Mn-induced neurotoxicity have been extensively studied, including oxidative stress, mitochondrial dysfunction, glutamate excitotoxicity, protein misfolding, inflammation, autophagy, mitophagy, endoplasmic reticulum stress, and apoptosis (Martinez-Finley et al., 2013; Harischandra et al., 2019a; Pajarillo et al., 2020a; Soares et al., 2020; Tinkov et al., 2021). Identifying the molecular targets, such as transcription factors (TFs) and intracellular proteins involved in Mn-induced neurotoxicity, could be critical for developing therapeutic strategies against Mn’s neurotoxicity. Several TFs have been found as potential molecular targets of Mn’s toxicity, namely RE1-silencing transcription factor (REST; neuron-restrictive silencer factor, NRSF) (Pajarillo et al., 2020a; Pajarillo et al., 2022a), Yin Yang 1 (YY1) (Karki et al., 2014a; Karki et al., 2015a), transcription factor EB (TFEB) (Zhang et al., 2020) and nuclear factor erythroid 2-related factor 2 (Nrf2) (Ijomone et al., 2022). Some intracellular proteins, particularly related to PD, were also dysregulated by Mn’s insults. These include leucine-rich repeat kinase 2 (LRRK2), PTEN-induced kinase 1 (PINK1), parkin, and α-synuclein (α-Syn) (Chen et al., 2018; Kim et al., 2019; Liu et al., 2021a; Liu et al., 2022a; Cao et al., 2022).

Numerous studies have been conducted in search of potential therapeutics for Mn’s toxicity, including antioxidants, anti-inflammatory, and anti-excitotoxic agents. Although the precise molecular targets of Mn-induced neurotoxicity are not fully understood, several agents have been shown to exhibit protective effects against Mn-induced neurotoxicity in both *in vivo* and *in vitro* models. Antioxidants are one of the most studied therapeutics against Mn-induced neurotoxicity. Accumulating evidence reveals that many natural and synthetic compounds inhibited Mn-induced oxidative stress (Bahar et al., 2017a; Ommati et al., 2020; Cong et al., 2021). Several compounds such as estrogens and riluzole attenuated Mn-induced glutamatergic excitotoxic neuronal injury by upregulating astrocytic glutamate transporters such as excitatory amino acid transporter 1 (EAAT1) and EAAT2 [glutamate-aspartate transporter (GLAST) and glutamate transporter 1 (GLT-1) in rodents, respectively] (Fumagalli et al., 2008; Lee et al., 2009a). A disaccharide trehalose attenuated Mn-induced neurotoxicity by modulating Mn-impaired autophagy and mitophagy (Liu et al., 2019; Jing et al., 2020). Chelating agents such as ethylenediaminetetraacetic acid (EDTA) (Discalzi et al., 2000) and para-aminosalicylic acid (PAS) have been shown to reduce acute Mn toxicity by promoting Mn excretion from the body (Jiang et al., 2006; Zheng et al., 2009). Intravenous EDTA chelation therapy has been clinically the primary treatment for manganism patients (for review, see Anagianni and Tuschl, 2019). However, several reports on its effectiveness have been controversial, and treatment outcome varies according to many factors, such as patient age, disease severity, and etiology (Tuschl et al., 2016; Lee and Shin, 2022). The lack of effective therapeutic approaches against Mn-induced neurotoxicity warrants the development of more efficacious drugs and targeted therapies.

**FIGURE 1:**
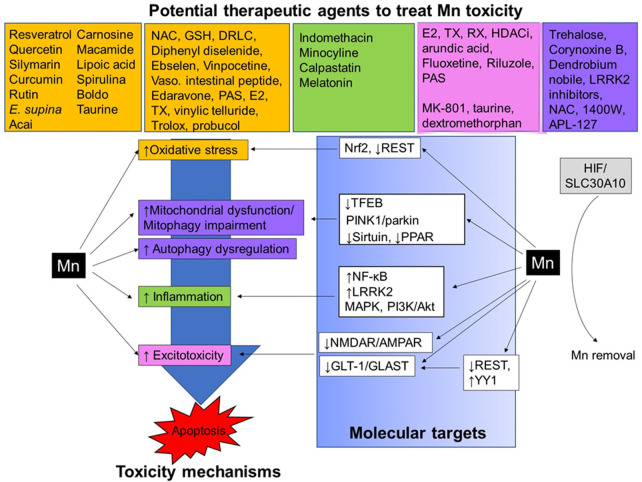
Neurotherapeutics and their proposed mechanism of action against Mn-induced neurotoxicity. Mn exposure causes several neurotoxic effects in the CNS, for example, oxidative stress, impairment of autophagy/mitophagy, mitochondrial dysfunction, inflammation, and excitotoxicity, leading to apoptotic cell death. Several TFs, including Nrf2, REST, TFEB, NF-κB, YY1, and HIF, as well as intracellular proteins such as sirtuin, LRRK2, PPAR, PINK1/parkin, MAPK, and PI3K/Akt, are involved in the regulation of these molecular mechanisms in Mn-induced neurotoxicity. Neurotherapeutic agents showing antioxidant (orange), anti-inflammatory (green), anti-excitotoxic (pink), and modulators of autophagy/mitophagy (purple) have demonstrated protective effects against Mn-induced neurotoxicity in *in vivo* and *in vitro* experimental settings.

In this review, we summarize the molecular mechanisms of Mn-induced neurotoxicity and potential molecular targets for neurotherapeutics (Figure 1). We also discuss protective agents with efficacy against Mn-induced neurotoxicity, such as antioxidants, anti-inflammatory agents, and modulators of glutamate transporters/receptors and autophagy/mitophagy. Understanding the mechanisms of Mn’s neurotoxicity associated with targeted modulators will greatly contribute to developing effective neurotherapeutics against manganism.

## 2 Mechanisms of Mn-induced toxicity in the brain

Multiple mechanisms of Mn-induced neurotoxicity have been reported and some of the established mechanisms of Mn toxicity are discussed in this review. The findings indicate that the dysregulations of these mechanisms may contribute to the onset and progression of manganism, potentially by decreasing gene expression of antioxidant enzymes, glutamate transporters, antiapoptotic proteins or increasing proinflammatory cytokine genes and proapoptotic proteins as well as impairing mitophagy/autophagy genes. Thus, understanding the regulation of molecular pathways involved in Mn toxicity may pinpoint target molecules that can modulate various pathways for neuroprotection.

### 2.1 Oxidative stress

Oxidative stress is implicated in a wide range of pathological processes, especially in neurodegenerative diseases such as PD and AD. Mn generated reactive oxygen species (ROS) and subsequent oxidative damage to neural cells by increasing ROS levels in the central nervous system (CNS) (Cordova et al., 2013; da Silva Santos et al., 2014; Maddirala et al., 2015). Mn caused oxidative stress in various regions of the rodent brain, particularly in the basal ganglia, such as globus pallidus, striatum, and substantia nigra (Ali et al., 1995; Dobson et al., 2003; Erikson et al., 2004). Mn-induced mitochondrial impairment significantly contributes to elevated ROS levels as Mn’s inhibition of mitochondrial ATP production increased electron leakage and ROS production (Scholte, 1988; Chen et al., 2001). Trivalent Mn^3+^, an oxidized form of Mn^2+^, induced higher oxidative damage, given that Mn^3+^ has a greater oxidative stress potential than that of Mn^2+^ (Ali et al., 1995; HaMai et al., 2001; Reaney and Smith, 2005). In addition to the direct production of ROS, Mn also dysregulated antioxidative defense systems (Szpetnar et al., 2016) by reducing the synthesis of the oxidative scavenger glutathione (GSH), thus depleting its levels and further exacerbating the oxidative insult of Mn (Yang et al., 2018).

### 2.2 Mitochondrial impairment and mitophagy

Mitochondria are dynamic organelles primarily for ATP production, and their dysfunction contributes to the incidences of neurodegenerative diseases. Upon entry into the cells, Mn preferentially accumulates within the mitochondria which are well-established targets of Mn-induced neurotoxicity (Alaimo et al., 2014; Sarkar et al., 2018). Mn disrupts mitochondrial homeostasis and alters mitochondrial bioenergetics (Gorojod et al., 2018), followed by the release of ROS, leading to a necrotic or apoptotic cellular death (Chen et al., 2016). Moreover, Mn promotes an imbalance in fission-fusion dynamics by dysregulating its associated proteins such as dynamin-related protein 1 (Drp-1), optic atrophy type 1(Opa-1), and mitofusin 2 (Mfn2) (Alaimo et al., 2014), leading to mitochondrial fragmentation and dysfunction (Morcillo et al., 2021). This Mn-induced mitochondrial impairment is also known to cause neuroinflammation (Sarkar et al., 2018).

Timely removal of damaged and dysfunctional mitochondria by mitophagy, a selective autophagy to eliminate damaged mitochondria for its quality control, protects against Mn-induced neurotoxicity, but the protective role of mitophagy is impaired upon prolonged exposure to Mn (Roberts et al., 2016; Shefa et al., 2019). Studies have shown that Mn induced S-nitrosylation of PINK1, resulting in the repression of PINK1/parkin-mediated mitophagy function, ultimately leading to cell damage (Liu et al., 2022a). Therefore, depending on the experimental conditions and settings, mitophagy function can be activated upon Mn exposure to protect cells by eliminating damaged mitochondrial proteins, while prolonged overexposure to Mn can impair PINK1/parkin-mediated mitophagy function, leading to cellular injury (Zhang et al., 2016; Huang et al., 2021).

### 2.3 Autophagy

Autophagy is responsible for the degradation of damaged organelles and macromolecules to maintain cellular homeostasis in response to cellular insults and stress (Ghavami et al., 2014). Following Mn exposure, this process is triggered initially to protect cells from cellular damage (Zhou et al., 2018; Porte Alcon et al., 2020). However, after prolonged exposure to Mn, autophagy function is impaired, and subsequent cytotoxicity occurs (Zhang et al., 2013), suggesting that pathological levels of Mn exposure dysregulated autophagy function. Studies have shown that Mn increased autophagy activation in the rat striatum within 4–12 h after Mn exposure, but Mn suppressed autophagy at later stages, 1–28 days post-treatment of Mn (Zhang et al., 2013). Similarly, Mn activated autophagy up to 12 h of its exposure in human SH-SY5Y cells, but Mn exposure over 24 h impaired autophagy (Ma et al., 2017). Mn-induced dysregulation of autophagy impaired other cellular organelles and activated the NLRP3-CASP1 inflammasome pathway in the hippocampus of mice and BV-2 cells, leading to hippocampal-dependent impairment in learning and memory (Wang et al., 2017). Mn injection into the striatum showed autophagy dysregulation in rats, characterized by an increased number of mitochondrial vacuoles, swollen and fragmented endoplasmic reticulum, and dysfunctional lysosomes (Bahr et al., 2012; Bryan et al., 2019; Zhang et al., 2020).

### 2.4 Inflammation

Astrocytes and microglia critically contribute to maintaining a homeostatic balance in the CNS by regulating immune responses with production of cytokines and inflammatory mediators. Studies have shown that Mn dysregulated mitochondrial bioenergetics in astrocytes, resulting in the release of proinflammatory cytokines and adjacent neuronal injury (Sarkar et al., 2018). Mn activated microglia, generating proinflammatory cytokines, leading to dopaminergic neuronal death in the rat brain (Zhao et al., 2009). Mn induced neuroinflammation by increased production and releases of proinflammatory factors such as TNF-α, interleukin (IL)-1β, and nitric oxide synthase 2 (NOS2) in microglia, leading to neuronal injury in non-human primates (Verina et al., 2011). The nuclear factor kappa-light-chain-enhancer of activated B cells (NF-κB) signaling pathway has been shown to play an essential role in inflammatory responses to Mn toxicity in microglia during Mn-induced release of microglial cytokines to further amplify inflammatory activation of astrocytes (Kirkley et al., 2017).

### 2.5 Glutamate excitotoxicity

Glutamate is the major excitatory neurotransmitter in the brain and involved in essential brain functions such as cognition, learning, and memory (Danbolt, 2001). However, excessive extracellular glutamate levels and, thus, overstimulating glutamate receptors induce excitotoxic neuronal injury. Studies have shown that Mn impaired the astrocytic glutamate transporter function and glutamate uptake, leading to disruption of glutamate homeostasis and excitotoxic neuronal injury (Erikson et al., 2006; Deng et al., 2012) as the astrocytic glutamate transporters, EAAT1 (GLAST in rodents) and EAAT2 (GLT-1 in rodents), are critical for the removal of synaptic glutamate to prevent excitotoxic neuronal injury. Numerous studies have shown that Mn decreased astrocytic glutamate uptake (Hazell and Norenberg, 1997; Normandin and Hazell, 2002; Mutkus et al., 2005) by downregulation of EAAT2 (GLT-1) and EAAT1 (GLAST) in non-human primate and astrocyte cultures (Erikson and Aschner, 2002; Erikson et al., 2002; Lee et al., 2009a). The mechanism involved in Mn-induced reduction in GLAST (EAAT1) and GLT-1 (EAAT2) was at the transcriptional level by increasing YY1 expression in the mouse brain as well as in astrocytes (Lee et al., 2012; Karki et al., 2014a; Johnson et al., 2018a; Johnson et al., 2018b; Pajarillo et al., 2018). Moreover, Mn increased the sensitivity of postsynaptic glutamate receptors to glutamate, ultimately leading to irreversible cell damage (Spadoni et al., 2000). Mn also decreased the expression of N-methyl-D-aspartate (NMDA) receptors in rats, which also contributed to Mn’s neurotoxicity in addition to the increased concentrations of extracellular glutamate (Xu et al., 2010a).

### 2.6 Apoptosis

Mn causes apoptosis by modulating several cellular events. Studies have shown that Mn activated apoptotic signal caspase-3 as a consequence of Mn-induced endoplasmic reticulum stress (Chun et al., 2001). Mn also activated caspase-3 and p53 signaling by increasing neuronal expression of K-homology splicing regulator protein (KHSRP) in rat striatum (Shi et al., 2015). Mn induced apoptosis by activating protein kinase R in PC12 cells (Yagyu et al., 2020) and decreasing expression of wild-type p53-induced phosphatase 1, which modulates p53 signaling in striatal neurons (Ma et al., 2015). Moreover, Mn-induced apoptosis is not solely dependent on mitochondrial impairment as it did not correlate with mitochondrial depolarization and the subsequent release of cytochrome C (Oubrahim et al., 2001).

### 2.7 Epigenetic modification

Epigenetic modifications, such as DNA methylation, histone modification, and microRNAs (miRNA), play a role in Mn-induced neurotoxicity as Mn-impaired epigenetic modifications dysregulated global gene expression, resulting in aberrant cellular responses and activities (for review, see Tarale et al., 2016). Studies have shown that Mn altered DNA methylation in mice and SH-SY5Y cells (Yang et al., 2016), modulating DNA methylation of 226 genes associated with disrupted mitochondrial integrity and cell cycle, and DNA damage response (Yang et al., 2016). Mn also increased DNA methylation of p53 gene, resulting in reduction of p53 transcription and increase of downstream expression of proinflammatory meditator COX-2 in microglia, while this Mn effect was abolished by a demethylation reagent 5-Aza-dC (Liu et al., 2022b).

Epigenetic modification *via* histones acetylation or deacetylation also contributes to Mn-induced neurotoxicity by modulation of histone acetyltransferase (HAT) and histone deacetylase (HDAC). HATs such as CREB-binding protein (CBP) or p300 generally promote transcription by enhancing histone acetylation, whereas HDACs such as HDAC1-8 and sirtuins reduce transcription by inhibiting access of transcription factors to DNA (Xia et al., 2020). It has been shown that Mn increased HDAC activity and decreased HAT, resulting in cellular injury in PC12 and SH-SY5Y cells (Guo et al., 2018). Mn-reduced CBP/p300, and its-enhanced HDAC activity contributed to Mn-reduced transcription of tyrosine hydroxylase (TH) in dopaminergic neurons (Pajarillo et al., 2020a). Mn activated several HDACs, such as HDAC1-5 in astrocytes (Karki et al., 2014a; Karki et al., 2015b; Pajarillo et al., 2020a; Pajarillo et al., 2020b; Rizor et al., 2022) as well as HDAC1 and sirtuins (class III HDAC) in neurons (Zhao et al., 2019; Sun et al., 2021). HDACs also contributed to Mn-induced decreases in expression of EAAT1 and 2 by enhancing their interactions with YY1, a transcriptional repressor of EAAT1/2 in astrocytes (Karki et al., 2015a), which was attenuated by treatment with HDAC inhibitors (HDACi) (Karki et al., 2014a). Indeed, HDACi have been shown to mitigate Mn’s toxic effects by normalizing HDAC activity, histone acetylation, and gene expression in the mouse brain and astrocytes (Karki et al., 2014a; Johnson et al., 2018a; Johnson et al., 2018b). Although the mechanism of Mn-induced dysregulation of histone acetylation warrants further investigation, abnormal histone modification is critically involved in Mn-induced neurotoxicity. Mn-induced oxidative stress contributes to Mn-induced dysregulation of histone modification as the antioxidant curcumin attenuated Mn-reduced histone acetylation at H3K18 and H3K27 and expression of antioxidant genes, such as SOD2 in rats (Yang et al., 2022).

Sirtuins are comprised of seven proteins (sirtuins 1–7), functioning as not only histone deacetylases but also signaling molecules such as ADP-ribosyl transferase (Flick and Luscher, 2012). Studies have shown that Mn decreased sirtuin 1 and 3 during Mn-induced neurotoxicity in PC12 cells, primary neurons, and mice (Zhao et al., 2019; Sun et al., 2021). Mn decreased sirtuin 1 with a concomitant increase in proinflammatory genes and NF-κB in microglia (Yan et al., 2021), while sirtuin 3 overexpression attenuated Mn-induced neurotoxicity in primary cortical neurons (Sun et al., 2021), suggesting that sirtuin 1 and 3 play a role in Mn-induced inflammation and neurotoxicity.

Mn could also regulate gene expression by altering miRNAs which are small single-stranded non-coding RNAs, dysregulating RNA expression and post-transcriptional expression. Mn altered the levels of 73 miRNAs in SH-SY5Y cells (He et al., 2017), including miR-4306, miR-7, and miR-433, which targets ATP13A2 (PARK9), α-Syn (SNCA), and fibroblast growth factor-20 (FGF-20), respectively, while inhibition of these miRNAs attenuated the Mn’s effects on those target genes in SH-SY5Y cells (He et al., 2017; Tarale et al., 2018). These indicate that miRNA regulation could be important targets for developing protection strategies against Mn’s toxic effects.

## 3 Potential molecular targets for neurotherapeutics to mitigate Mn’s neurotoxicity

Dysregulations of Mn-induced cellular processes are associated with altering Mn’s target genes at the molecular and transcriptional levels. In this section, therefore, we will update findings of Mn-modulated genes, such as TFs and intracellular proteins, which could contribute to the development of strategies for therapeutics against Mn-induced neurotoxicity. These potential targets have shown promising attributes that can mitigate the serious harmful effects and neurological deficits caused by Mn toxicity.

### 3.1 TFs

Given the critical role of cellular insults such as mitochondrial dysfunction, oxidative stress, inflammation, and excitotoxicity in Mn-induced neurotoxicity, Mn’s target genes that are dysregulated and involved in those impaired cellular processes have been identified. Mn has been shown to modulate several TFs such as REST, YY1, Nrf2, TFEB, hypoxia-inducible factor 1α (HIF1α), NF-κB, and peroxisome proliferator-activated receptor gamma (PPAR-γ) in both *in vitro* and *in vivo* settings. The following proceedings discuss each of Mn’s target genes for their mechanistic roles in Mn-induced neurotoxicity and the potential for targeting specific molecules to treat Mn-induced neurotoxicity.

#### 3.1.1 REST

REST is a zinc-finger TF, playing a critical role in neurogenesis, differentiation, growth, stress response, and survival by binding to the DNA sequence motif known as repressor element 1 (RE1, aka NRSE) (Chong et al., 1995; Schoenherr and Anderson, 1995). REST is increased in normal aging brains to maintain neuronal function by mitigating oxidative stress and apoptosis in aged human brains (Lu et al., 2014; Pajarillo et al., 2020a). Accordingly, REST was dysregulated in neurodegenerative diseases such as AD and PD (Lu et al., 2014; Pajarillo et al., 2020a). REST is expressed in various neural cell types, such as neurons, astrocytes, and microglia, implicating its diverse roles *via* different neural cells in the brain (Pajarillo et al., 2021; Pajarillo et al., 2022a). Mn decreased REST expression in dopaminergic neurons and astrocytes (Pajarillo et al., 2021; Pajarillo et al., 2020a), suggesting that impairing REST signals in neural cells might be involved in Mn toxicity mechanisms. Overexpression of REST inhibited Mn-increased pro-inflammatory cytokines such as TNF-α and proapoptotic proteins such as Bax, as well as attenuated Mn-decreased antiapoptotic Bcl-2 and antioxidant Nrf2, heme oxygenase 1 (HO-1), and catalase (CAT) in dopaminergic neurons (Pajarillo et al., 2020a). REST also attenuated the Mn-induced reduction of TH, a rate-limiting enzyme for dopamine synthesis in dopaminergic neurons (Pajarillo et al., 2020a).

Moreover, Mn decreased REST expression in astrocytes, while overexpression of astrocytic REST exerted protection against Mn-induced neuronal cell toxicity by regulating inflammatory cytokines and glutamate transporter GLT-1/EAAT2 in astrocytes (Pajarillo et al., 2021). Mn also reduced REST with a concomitant decrease in GLT-1 in the mouse striatum (Pajarillo et al., 2022a). REST has been shown to upregulate EAAT2 at the transcriptional levels by its binding to the RE1 sites of the EAAT2 promoter in astrocytes (Pajarillo et al., 2021). Conversely, downregulation of REST further decreased Mn-reduced EAAT2 in astrocytes and excitotoxic damage to dopaminergic neuronal cells in astrocyte-neuron co-culture (Pajarillo et al., 2021). Deletion of astrocytic REST in the mouse striatum exacerbated Mn-reduced TH protein levels and behavioral deficits (Pajarillo et al., 2022a). REST’s protective mechanisms against Mn-induced neurotoxicity were also associated with repressing inflammatory cytokines (McGann et al., 2021), at least in part, by its direct binding to their promoters and regulating gene expression (McGann et al., 2021; Pajarillo et al., 2022a).

#### 3.1.2 YY1

YY1 is a TF, playing a dual role in transcription of its target genes, regulating both transcriptional activation and repression in a context- or cofactor-dependent manner (Gordon et al., 2006). YY1 in both neuronal and non-neuronal cells is critical for normal brain development and function (for review, see Rylski et al., 2008). Astrocytic YY1 regulates oxidative stress, inflammation, and apoptosis in the mouse brain (Pajarillo et al., 2022b), and Mn increases YY1 *via* NF-κB signaling and promotes YY1 binding to the GLT-1/GLAST promoters, resulting in repression of GLT-1/GLAST expression and function in astrocytes (Karki et al., 2014a; Pajarillo et al., 2020b). This Mn effect on YY1 could be closely associated with Mn-induced excitotoxic neuronal injury as reduction of GLT-1/GLAST would accumulate glutamate in the synaptic clefts and overstimulate its receptors. Deletion of astrocytic YY1 in substantia nigra of the mouse brain attenuated the Mn-induced decrease in GLAST and GLT-1 protein levels as well as TH levels in the nigrostriatal pathway, resulting in ameliorating Mn-induced motor deficits (Pajarillo et al., 2020b). These findings suggest that YY1 could be a potential molecular target in mitigating Mn-induced neurotoxicity.

#### 3.1.3 Nrf2

Nrf2 is a transcriptional regulator of genes involved in antioxidant enzymes, including HO-1, glutathione peroxidase (GPx) 2, glutathione S-transferases, and thioredoxin by binding to its consensus site, antioxidant response element (ARE), of target genes to mitigate oxidative toxic insults (Tonelli et al., 2018). Mn dysregulated Nrf2-superoxide dismutase (SOD), along with inducing oxidative stress by increasing levels of isoprostanes and neuroprostanes in the rat brain (Santos et al., 2012). Mn decreased HO-1 expression by dysregulating Nrf2 activity in PC12 cells (Li et al., 2011a), while enhancing the Nrf2-HO-1 pathway protected neurons and astrocytes against Mn-induced toxicity (Gorojod et al., 2018; Pajarillo et al., 2020a). HO-1 overexpression also attenuated Mn-induced mitochondrial damage, oxidative stress, and cytotoxicity in C6 cells (Gorojod et al., 2018). Mn downregulated Nrf2 by modulating its transcription and degradation mechanisms *via* the ubiquitination-proteasome pathway (Li et al., 2011b; Pajarillo et al., 2020a). Mn-induced Nrf2 modulation was region-as well as sex-specific in rat brain (Ijomone et al., 2022). Histone deacetylation was also involved in Mn’s downregulation of the Nrf2 pathway in PC12 cells (Zhang et al., 2017), suggesting that Mn dysregulates Nrf2 *via* various intracellular targets.

#### 3.1.4 TFEB

TFEB regulates genes involved in autophagy, lysosomal degradation, lipid catabolism, energy metabolism, and immune response (Settembre et al., 2011). Studies have shown that Mn impaired autophagy-lysosomal degradation and mitochondrial function by hampering TFEB translocation into the nucleus in astrocytes (Zhang et al., 2020). The mechanism of these Mn effects on TFEB is partly by activation of the MAPK1/ERK2 and MAPK3/ERK1 pathways to phosphorylate TFEB, resulting in its sequestration in the cytosol as an inactive form (Settembre et al., 2012). TFEB overexpression attenuated Mn-reduced autophagy-related TFEB target genes such as Lamp1, and Wipi1 (Zhang et al., 2020), restoring Mn-dysregulated autophagy flux and mitochondrial function in astrocytes.

#### 3.1.5 HIF-1 and HIF-2

HIF-1 and 2 are TFs, playing a significant role in proliferation, oxidative stress, inflammation, and apoptosis, by binding to its hypoxia response elements (HRE) on the promoter region of its target genes under hypoxic or toxic conditions (Chapman-Smith et al., 2004). Upregulation of HIF increased expression of Mn exporter SLC30A10 in the liver and induces protection against Mn’s neurotoxic effects in mice (Liu et al., 2021b). Moreover, Mn itself increased expression of HIF-1α/2α and SLC30A10, possibly to excrete excess Mn as a homeostatic mechanism. These findings suggest that HIF-SLC30A10 could be a relevant target to eliminate excess Mn for maintenance of Mn homeostasis in the body and prevent Mn’s neurotoxicity.

#### 3.1.6 NF-κB

NF-κB, a master regulator of genes involved in cytokine production and cell survival, plays an important role in Mn-induced neurotoxicity (for review, see Pajarillo et al., 2021). Mn activates NF-κB to enhance expression of genes involved in the production of various proinflammatory cytokines and chemokines in astrocytes and microglia (Barhoumi et al., 2004; Filipov et al., 2005; Prabhakaran et al., 2008). NF-κB plays a role in cell-to-cell communication signaling for transferring inflammatory products between astrocytes and microglia during Mn toxicity (Kirkley et al., 2017; Nkpaa et al., 2019; Li et al., 2021). NF-κB was involved in Mn-induced production of cytokines IL-1β/IL-18 *via* enhancing NLRP3 inflammasome formation in BV-2 microglia (Peng et al., 2020). Mn also activated NF-κB to increase YY1 expression, which in turn, repressed GLT-1 (EAAT2) in astrocytes (Karki et al., 2014a). These findings indicate that the NF-κB pathway is involved in various Mn toxicity mechanisms, providing the potential to be a therapeutic target to treat Mn’s neurotoxicity.

#### 3.1.7 PPARs

PPARs are TFs with three isotypes, PPAR-α, PPAR-β/δ, and PPAR-γ, playing a role in development, proliferation, differentiation, lipid and energy metabolism, and survival (Desvergne and Wahli, 1999; Michalik et al., 2004). Studies have shown that Mn dysregulated cellular localization and protein levels of PPARs in human U87 astrocytes and SK-N-SH neuronal cells (Isaac et al., 2006). PPAR-γ activation attenuated Mn-induced loss of cell viability, oxidative stress, and mitochondrial damage in U87 astrocytes (Gugnani et al., 2018).

### 3.2 Mn’s intracellular target proteins

Intracellular communications such as signaling pathways are dysregulated in Mn-induced toxicity. Several intracellular proteins such as PINK1, parkin, LRRK2, and α-Syn are modulated under Mn-induced neurotoxicity, suggesting that these proteins might be potential molecular targets to develop therapeutics against Mn-induced neurotoxicity.

#### 3.2.1 PINK1

PINK1, is an important mediator of mitophagy in regulation of mitochondrial quality control and damaged mitochondrial protein degradation with collaboration of parkin, and its mutations are associated with familial forms of PD (Springer and Kahle, 2011). Studies have shown that α-Syn interfered with PINK1’s mitophagy function in Mn-exposed rat brain (Liu et al., 2021a), accompanied by reduction in ATP levels and mitochondrial membrane potential, but increased mitochondrial ROS levels in the striatum. However, knockdown of α-Syn enhanced PINK1 activity and mitophagy and attenuated Mn-induced oxidative stress, mitochondrial dysfunction, and cell death in SH-SY5Y cells (Liu et al., 2021a).

Mn increased S-nitrosylation of PINK1, resulting in impaired mitophagy and thus, accumulating damaged mitochondria and cell death in the rat striatum and primary neurons (Liu et al., 2022a). Mn also increased PINK1 DNA methylation in SH-SY5Y cells (Tarale et al., 2017), leading to a decrease in PINK1 mRNA expression (Tarale et al., 2017). These findings indicate that Mn dysregulates PINK1 by multiple mechanisms in Mn-induced impairment of mitophagy and consequential neuronal injury.

#### 3.2.2 Parkin

Parkin, an E3 ubiquitin ligase encoded by the PARK2 gene, is associated with familial PD as its mutations lead to its loss-of-function in mitophagy (Seirafi et al., 2015). Mn decreased parkin mRNA and protein levels in the striatum and midbrain under Mn-induced dopaminergic neurotoxicity and motor deficits in rats (Cao et al., 2022), while overexpression of parkin attenuated Mn-induced cell death in SH-SY5Y cells (Higashi et al., 2004), indicating that parkin exerts protective effects against Mn’s neurotoxicity. Other studies have reported that Mn increased parkin expression in rats (Sriram et al., 2010) and phosphorylation of parkin in the striatum of rats (Liu et al., 2021a). This Mn’s effect on increasing parkin might attribute to a compensatory mechanism to cope with Mn’s toxicity in mitophagy by enhancing mitophagy in subtoxic conditions. While parkin is directly involved in Mn-impaired mitophagy, it could also play critical functions in Mn accumulation during Mn-induced dopaminergic neurotoxicity. Dysfunction or mutation of parkin resulted in Mn accumulation (Bornhorst et al., 2014), possibly by downregulating Mn transporter ferroportin in *C. elegans* (Chakraborty et al., 2015). Overexpression of parkin prevented Mn accumulation by reduction of divalent metal transporter 1 (DMT1, responsible for Mn uptake) *via* ubiquitin-proteasomal degradation in SH-SY5Y cells (Roth et al., 2010), and deletion of parkin did not alter tissue Mn levels compared to WT mice after Mn exposure (Foster et al., 2018). These mixed results may be due to different experimental models and settings, requiring further studies to understand parkin’s role in Mn transport and accumulation.

#### 3.2.3 LRRK2

LRRK2 is a large multi-domain protein, composed of a leucine-rich domain (LRR), a Roc GTPase domain, a carboxy-terminal of Ras (COR) domain, a kinase domain and a WD40 domain at the carboxy-terminal (Dzamko, 2017). Several mutations in LRRK2 are associated with sporadic PD (accounting for 1-2% of all sporadic PD cases) and a familial PD (up to 13% of all familial PD cases) (Healy et al., 2008). Among all identified pathological LRRK2 mutations, G2019S is the most prevalent mutation in both familial and sporadic PD cases (Healy et al., 2008). Most pathogenic LRRK2 mutations, such as G2019S and R1441 C/G/H, enhances LRRK2 kinase activity by two-to three-fold (Dzamko, 2017). Mn increased both LRRK2 expression and its kinase function, which played a critical role in Mn-induced autophagy, oxidative stress, and inflammation (Chen et al., 2018; Kim et al., 2019). LRRK2 inhibition attenuated Mn-induced autophagy-related proteins Beclin 1 and Atg5, along with inhibition of Mn-induced apoptosis and TNF-α production in microglia (Chen et al., 2018; Kim et al., 2019).

#### 3.2.4 α-Syn

Mn increased α-Syn expression, aggregation, and subsequent cytotoxicity in various experimental models (Verina et al., 2013; Xu et al., 2013; Bhandari et al., 2018), while knockdown of α-Syn attenuated Mn-induced neurotoxicity (Cai et al., 2010; Li et al., 2010), indicating that α-Syn plays a critical role in Mn-induced cytotoxicity. Mn also modulated the secretion and extracellular vesicular trafficking of α-Syn between cells by activating RAB27A for exosomal packaging of α-Syn (Pfeffer, 2010), resulting in the exosomal release into the extracellular environment (Harischandra et al., 2018). Welders chronically exposed to Mn increased misfolded α-Syn in their exosomes from their serum samples, which have been shown to induce neuroinflammation and subsequent neurodegeneration (Harischandra et al., 2019b).

#### 3.2.5 PI3K/Akt

PI3K is a group of signaling proteins that perform diverse cellular functions such as cell growth, proliferation, differentiation, survival, autophagy, and intracellular trafficking. PI3K activates other intracellular kinases, including Akt and the mammalian target of rapamycin (mTOR), to modulate various cellular pathways. Mn activated PI3K to induce inducible NOS (iNOS) expression in BV2 microglia (Bae et al., 2006). Mn also activated the PI3K/Akt pathway in Mn-induced apoptosis, such as caspase-3 and Bax levels in rat hippocampus (Cheng et al., 2018), indicating PI3K/Akt signaling is involved in Mn-induced apoptosis. In contrast, enhancing PI3K activity induced protection mechanisms against Mn toxicity as inhibition of PI3K/Akt increased Mn’s cytotoxicity and reduced antioxidant enzymes in PC12 cells (Tan et al., 2022). In fact, many studies support the role of PI3K/Akt signaling in protective mechanisms against Mn-induced toxicity (Ji et al., 2020). 17β-estradiol (E2) and tamoxifen (TX) attenuated Mn-induced oxidative stress and EAAT2 dysregulation *via* the PI3K/Akt signaling pathway in astrocytes (Lee et al., 2009a). While Mn activates the PI3K/Akt pathway, further investigation is required to better understand its distinct roles in Mn-induced neurotoxicity and neuroprotection.

#### 3.2.6 MAPK

MAPK are serine and threonine protein kinases that are essential in regulating brain development, gene transcription, protein synthesis, proliferation, differentiation, metabolism, apoptosis, and cell-cell interaction (Cargnello and Roux, 2011). Mn has been shown to activate MAPK/ERK and p38 signaling pathways in inducing oxidative stress and inflammation in astrocytes and microglia (Crittenden and Filipov, 2008; Moreno et al., 2008). MAPK/ERK signaling may be involved in modulating the transcription of genes such as iNOS to attenuate Mn-increased oxidative stress and inflammation in BV2 microglia (Bae et al., 2006). Mn also induced astrocyte swelling and damage *via* MAPK signaling, as well as protein levels of aquaporins in primary astrocytes (Rao et al., 2010). The Mn-induced activation of the MAPK pathway exacerbated AD pathogenesis as Mn induced hyperphosphorylation of tau by activating the MAPK/ERK pathway in PC12 cells (Cai et al., 2011). MAPK/ERK signaling also appears to be activated by both Mn-induced toxicity mechanisms and protection induced by various agents against Mn toxicity, warranting further studies.

## 4 Neurotherapeutics and their potential underlying mechanisms against Mn’s neurotoxicity

Several pharmacological agents have been shown to exert protective effects against Mn-induced neurotoxicity by attenuating Mn-induced mitochondrial impairment, oxidative stress, inflammation, glutamate transporter dysregulation, and apoptosis in various *in vitro* and *in vivo* models. Despite the significant efficacy, their precise molecular mechanisms associated with mitigating Mn-induced neurotoxic effects remain to be elucidated. Various pharmacological agents’ potential molecular targets and their mode of action in attenuating Mn-induced neurotoxicity are summarized (Tables 1, 2).

**TABLE 1:** Neurotherapeutic agents and their protective effects against Mn-induced oxidative stress in *in vitro* and *in vivo* experimental settings.

Therapeutic agent	Concentration/Dosage	Mode of action or effects	Type of study (neural cell type, if applicable)	Mn concentration/dosage	References
***Antioxidant***					
***Natural compounds***					
Resveratrol	30 mg/kg, po	↓ROS level, ↑SOD activity, ↑CAT activity, ↔GSH level, ↑GSH/GSSG, ↓MDA, ↑Nrf2 ↑HO-1	mice (male)	200 µmol/kg, po	Cong et al. (2021)
		↓TNF-α, ↓p-NF-κB p65, ↓IL-1β, ↓COX-2, ↓p-STAT3, ↓p-JNK			
		↔SIRT1 activity, mRNA, protein levels			
	10 and 20 mg/kg, po	↓GSSG/GSH, ↑SOD activity, ↑α-tocopherol levels	rats (male)	20 mg/kg, po	Gawlik et al. (2017)
	10 and 20 µM	↓mitochondrial ROS, ↓mitochondrial damage, ↑SIRT1, ↑PGC1α, ↑SIRT3, ↑NRF1, ↑TFAM	primary cortical neurons	100 and 200 µM	Sun et al. (2021)
		↓apoptosis, ↓c-caspase-3, ↓cytochrome c, ↓Bax, ↑Bcl-2,			
	30 µM	↓apoptosis, ↑Bcl-2, ↑PUMA, ↓FOXO3a, ↑SIRT1	*In vitro*, PC12 cells ; *in vivo*, mice (male)	500 µM	Zhao et al. (2019)
Quercetin	13 and 26 mg/kg, po	↑SOD activity	Rats (male)	20 mg/kg, po	Gawlik et al. (2017)
	10 and 20 mg/kg	↔SOD activity, ↓CAT activity, ↓MDA, ↓H2O2 levels	Rats (male)	15 mg/kg, po	Adedara et al. (2017)
	*In vitro* (10 and 20 µg/ml); *in vivo* (25 and 50 mg/kg, po)	↑Nrf2, ↑HO-1, ↓ROS, ↓MDA, ↓protein carbonyl, ↔SOD activity, ↔CAT activity, ↔GSH level, ↔mitochondrial potential, ↓apoptosis	*In vitro*, SK-N-MC); *in vivo*, rats (male)	*In vitro* (500 µM); *in vivo* (15 mg/kg, ip)	Bahar et al. (2017)
		TNF-α, COX-2, iNOS, IL-1β, IL-6, Bax, Bcl-2. Cytochrome c, c-caspase-3, PARP-1, ↓NF-κB p65, ↓p-IκB			
Silymarin	100 mg/kg, ip	↔SOD activity, ↔GPx activity, ↔CAT activity↓H2O2 levels	rats (male)	20 mg/ml (100 mM of Mn^2+^), po	Chtourou et al. (2010)
Curcumin	0.1-10 µM	↓MDA, ↑HO-1, ↔ROS levels, ↔cell viability, ↓c-caspase-3, ↓cytosolic cytochrome c, ↓Bax, ↑Bcl-2, ↑mitochondrial potential	BV-2 cells	250 µM	Park and Chun (2017)
	400 mg/kg, po	↓MDA, ↔CAT, ↔SOD1, ↔SOD2, ↓H3K18 acetylation, ↑H3K27 acetylation, ↔TH levels	rats (male)	5, 10, 15 mg/kg, ip	Yang et al. (2022)
Rutin	50 and 100 mg/kg, po	↔SOD activity, ↔CAT activity, ↔GPx activity, ↓TNF-α, ↓IL-1β, ↓IL-6	rats (male)		Nkpaa et al. (2019)
Euphorbia supina	100 and 200 µg/mL (*in vitro*); 100 and 200 mg/kg (*in vivo*), po	↓ROS, ↓MDA, ↔GSH, ↔SOD, ↔CAT	*In vitro*, SK-N-MC cells; *in vivo*, rats (male)	500 µM (*in vitro*); 15 mg/kg (*in vivo*), ip	Bahar et al. (2017)
		↔mitochondrial potential, ↑cell viability, ↓apoptosis			
Acai (Euterpe oleracea Mart.)	0.1 µg/ml	↑GSH/GSSG, ↑Glutamate uptake, ↓lipid peroxidation, ↓Nrf2	primary astrocytes	500 µM	da Silva Santos et al. (2014)
Carnosine	10, 50, 100 mg/kg, ip	↓ROS production, ↑GSH levels, ↑GSH/GSSG, ↑total antioxidant capacity, ↓GSSG levels, ↓lipid peroxidation	mice (male)	100 mg/kg, sc	Ommati et al. (2020)
		↑mitochondrial dehydrogenase activity, cell viability, ATP, ↓mitochondrial depolarization			
		↓mitochondrial swelling			
Macamide	0.1-50 µM	↑GSH, ↑mitochondrial potential, ↔cell viability	human primary glioblastoma U-87 MG	10 µM	Gugnani et al. (2018)
		↑PPARγ level			
Lipoic acid	50 mg/kg, gv	↔MDA levels, ↔GPx	rats (male)	74 mg/kg, gv	Ibrahim et al. (2020)
Spirulina platensis	300 mg/kg, gv	↔MDA levels, ↔GPx	rats (male)	74 mg/kg, gv	Ibrahim et al. (2020)
Boldo (Peumus boldus) extract	5 mg/mL	↓thiobarbituric acid reactive substances, ↓mortality	Drosophila melanogaster	3 mM	Bianchini et al. (2016)
Taurine	0.1, 1 and 10 mM	↓ROS production	*In vitro*, mouse brain mitochondria	0.01 mM-10 mM	Ahmadi et al. (2018)
		↑mitochondrial dehydrogenase activity, cell viability, ↑ATP, ↓mitochondrial swelling			
		↓mitochondrial depolarization			
	50, 100, 500 mg/kg, ip	↓ROS production, ↑GSH levels, ↑GSH/GSSG, ↑total antioxidant capacity, ↓GSSG levels, ↓lipid peroxidation	mice (male)	100 mg/kg, sc	Ommati et al. (2019)
		↑mitochondrial dehydrogenase activity, cell viability, ATP, ↓mitochondrial depolarization, ↓mitochondrial swelling			
***Synthetic compounds***					
N-acetylcysteine	1 mM	↓oxidative DNA damage, ↓Annexin/PI apoptosis	SH-SY5Y cells	2 µM and 62 µM	Stephenson et al. (2013)
	750 µM	↓lipid peroxidation, ↓ROS level, ↔glutathione reductase ↔ glutathione peroxidase, ↔mitochondrial potential, ↔ATP level	SH-SY5Y cells	800 µM	Maddirala et al. (2015)
		↑cell viability			
	100 mg/kg, ip	↓ROS level, ↓MDA, ↓nitric oxide	rats	25 mg/kg, ip	Chopra et al. (2021)
		↑GSH, ↑GSH/GSSG, ↓GPx, ↑GST, ↑GR, ↑SOD, ↑CAT			
Glutathione	1 mM	↓oxidative DNA damage, ↓Annexin/PI apoptosis	SH-SY5Y cells	2 µM and 62 µM	Stephenson et al. (2013)
Diphenyl diselenide	10 and 20 µmol per kg	↔total thiol level, ↔glutathione-S-transferase, ↔CAT, ↓ROS/RNS levels, ↓thiobarbituric acid reactive substances, ↓mortality	Drosophila melanogaster	30 mmol per kg	Adedara et al. (2016)
Ebselen	15 mg/kg, ip	↓E2 prostaglandin, ↔GSH level, ↓prolactin	rats (male)	10 mg/kg, ip	Santos et al. (2012)
Vinpocetine	6 mg/kg, po	↓MDA, ↑total antioxidant capacity, ↑SOD activity, ↑mitochondrial potential	rats (male)	25 mg/kg, ip	Nadeem et al. (2018)
		↓TNF-α, ↓IL-1β, ↓caspase-3			
	1-100 µM	↔cell viability, ↓LDH, ↔mitochondrial function, ↔ATP, ↓ROS, ↔Bax	NE-4C cells	2-250 µM	Bora et al. (2016)
Vasoactive intestinal peptide	0.03-1 µM	↔cell viability, ↓LDH, ↔mitochondrial function, ↔ATP, ↓ROS, ↔Bax	NE-4C cells	2-250 µM	Bora et al. (2016)
D-Ribose-L-Cysteine (DRLC)	200 mg/kg, po/ip	↑CAT activity, ↑SOD activity, ↓lactate dehydrogenase activity	rats (male)	25 mg/kg, ip (2 weeks)	Akingbade et al. (2022)
Edaravone	1 and 5 mg/kg, im	↓lipid peroxidation, ↔mitochondrial function, ↓protein carbonyls/oxidation	rats (male)	10 mg/kg, ip	Apaydin et al. (2016)
	10, 100 and 1000 µM	↓lipid peroxidation, ↔cell viability, ↓apoptosis	primary rat astrocytes	500 µM	Evren et al. (2015)
Para-amino salicylic acid (PAS)	120 mg/kg, sc	↔GSH level, ↓neuroprostanes, ↓prolactin	rats (male)	10 mg/kg, ip	Santos et al. (2012)
Dithiolethione ACDT	75 µM	↓ROS level, ↑GSH level, ↑NQO1, ↔cell viability	SH-SY5Y cells	300 µM	Kulkarni et al. (2021)
17β-estradiol (E2)	0.25 mg/pellet, 21-d release, neck implant	↔CAT levels and activity, ↔GSH level, ↓MDA	mice (ovx female)	0.4 µl of 1 µmol/µl of MnCl_2_, direct striatal injection	Pajarillo et al. (2018)
		↑GLAST/GLT-1 expression, ↔Bax, ↑Bcl-2			
Tamoxifen (TX)	5 mg/pellet, 21-d release, neck implant	↔CAT levels and activity	mice (ovx female)	0.4 µl of 1 µmol/µl of MnCl_2_, direct striatal injection	Pajarillo et al. (2018)
		↔GSH level, ↓MDA			
		↑GLAST/GLT-1 level, ↔Bax, ↑Bcl-2			
Diethyl-2-phenyl-2-telluropheny vinyl phosphonate	0.15 µmol/kg, ip	↔MDA, ↔mitochondrial function, ↔glutamate levels	rats (male)	13.7 mg/kg, po	Avila et al. (2010)
	1, 10 and 50 µM	↓ROS, ↓protein carbonyl	*C. elegans*	35 mM	Avila et al. (2010)
Trolox	1 mg/kg, ip	↓F2-isoprostanes, ↔p-p38, ↓caspase activity	rats (male and female)	5, 10, 20 mg/kg, ip	Cordova et al. (2013)
	100 µM	↓F2-isoprostanes, ↔ATP levels	primary rat neurons	500 µM	Milatovic et al. (2011)
Probucol	1, 5, 10 µM	↔mitochondrial health, ↓ROS, ↔cell viability	SH-SY5Y cells, C6 cells	100 µM and 500 µM	da Silva et al. (2022)

ip, intraperitoneal; sc, subcutaneous; po, per oral; gv, oral gavage; im, intramuscular; ovx, ovariectomized; ROS, reactive oxygen species; MDA, malondialdehyde; GPx, glutathione peroxidase; GSSG, glutathione disulfide; GSH, glutathione; SOD, superoxide dismutase; CAT, catalase; TNF-α, tumor necrosis factor alpha; IL-1β, interleukin-1β; Bcl-2, B-cell lymphoma 2; Bax, Bcl-2 associated X protein; COX-2, cyclooxygenase-2; HO-1, heme oxygenase 1; Nrf2, nuclear factor erythroid 2-related factor 2; NQO1, NADPH:quinone oxidoreductase-1; SIRT, sirtuin; PGC1α, PPARγ, coactivator-1α; PUMA, p53-up-regulated-modulator of apoptosis.

**TABLE 2:** Neurotherapeutic agents and their protective effects against Mn-induced inflammation, excitotoxicity and dysregulation of autophagy and mitophagy in *in vitro* and *in vivo* experimental settings.

Therapaeutic agent	Concentration/Dosage	Mode of action or effects	Type of study (neural cell type, if applicable)	Mn concentration/dosage	References
**Anti-inflammatory**					
Indomethacin	100 µM	↓F2-isoprostanes, ↔ATP levels	primary rat neurons	500 µM	Milatovic et al. (2011)
Minocycline	20 mg/kg, ip	↓Macrophage/microglia number	rats	25 and 50 µg/µL, intranigral injection	Ponzoni (2012)
		↔ DAergic neuronal loss			
Calpain inhibitors (calpastatin)	5 µg/kg, in	↓IL-1β, ↓TNF-α, ↓NFκB (hippocampus & stratum), ↓Iba1 (straitum)	rats (male)	5 mg/kg, in	Ivleva et al. (2022)
Melatonin	10 µM	↓IL-1β, ↓TNF-α, ↑IκB-α, ↓NF-κB, ↓iNOS, ↓NO	BV-2 cells	100 µM	Park and Chun (2017)
**Glutamate transporter modulators**					
Estrogen & SERM					
E2	E2 20 nM, TX 1 µM	↑EAAT1/GLAST, ↑EAAT2/GLT-1, ↑TGF-β1 via PI3K-Akt, MAPK/ERK	primary cortical astrocytes	250 µM	Lee et al. (2009)
	E2 10 nM, TX 1 µM	↑EAAT2/GLT-1 via TGF-α signaling,	primary rat cortical neurons	250 µM	Lee et al. (2012)
	E2, 400 mg/kg (0.25 mg/21-day release, neck implant)	↑GLAST, ↑GLT-1, ↑TGF-α, ↑ER-α, ↑CAT, ↑GSH, ↓MDA, ↓Bax/Bcl-2	mice (ovx female)	0.4 µl of 1 µmol/µl of MnCl_2_, direct striatal injection	Pajarillo et al. (2018)
	TX, 5 mg/pellet, 21-d release, neck implant				
TX	E2 20 nM, TX 1 µM	↑EAAT1/GLAST, ↑EAAT2/GLT-1, via PI3K-Akt, TGF-α, ERK, NF-κB, ↑nuclear ER binding	*in vitro*, primary cortical astrocytes; *in vivo*, mice	250 µM	Lee et al. (2009); Pajarillo et al. (2018)
Raloxifene (RX)	RX 1 µM	↑GLT-1 via ERK, EGFR, and CREB mediated by nuclear ER binding, ↑GLAST via nuclear ER binding and NF-κB	rat primary cortical astrocytes, rat primary neurons	250 µM	Karki et al. (2014)
HDACi					
Valproic Acid (VPA)	200 mg/kg, ip	↑GLT-1, ↑GLAST	*in vitro*, H4 astrocytes; *in vivo*, mice (male)	30 mg/kg, in	Johnson et al. (2018)
VPA & Sodium Butyrate (NaB)	VPA: 200 mg/kg, ip, NaB: 1200 mg/kg, ip	↑GLT-1, ↑GLAST	mice (male)	30 mg/kg, in	Johnson et al. (2018)
Arundic acid	50 µM	↑EAAT1/ via NF κB, ERK, and Akt pathways	H4 astrocytes and human primary astrocytes	250 µM	Karki et al. (2018)
**Glutamate receptor modulator**					
Riluzole	21.35 µmol/kg, sc	↔ GLT-1, GLAST	rats	200 µmol/kg, ip	Deng et al. (2009)
		↔ GS, PAG, Na+-K+-ATPase activity			
	100 μM	↔Glu uptake, ↔Na+-K+ ATPase ↔GS, ↑GLAST, ↑GLT-1, and ↑GS mRNA	primary astrocytes	500 μM	Deng et al. (2012)
	*In vitro*, 100 μM; *in vivo* 32 μmol/kg, sc	↓ EphrinA3, ↓ Glu, ↑ Na+-K+ ATPase activity, ↑GLT-1, ↑GLAST	*In vitro*, primary mouse striatal astrocytes; *In vivo*, mice	*In vitro*, 500 μM; *in vivo*, 50 mg/kg, ip	Qi et al. (2020)
Fluoxetine	*In vitro*, 100 μM; *in vivo*, 15 mg/kg, sc	↓ EphrinA3, ↓ Glu, ↑ Na+-K+ ATPase activity, ↑GLT-1, ↑GLAST	*In vitro*, primary mouse striatal astrocytes; *In vivo*, mice	*In vitro*, 500 μM; *in vivo*, 50 mg/kg, ip	Qi et al. (2020)
**Autophagy and mitophagy modulators**					
**Natural compounds**					
Trehalose	2% and 4% (w/vol), po	↑autophagolysosomes, ↑LC3-II/LC3-I, ↓p62	mice	200 µmol/kg, ip	Jing et al. (2020)
		↔apoptosis, ↓ROS, ↑SOD activity, ↓MDA level, ↓αSyn oligomers			
	2% and 4% (w/vol), po	↑PARP-cleaved, ↑autophagic vacuoles, ↑LC3-II/LC3-I, ↓p62, ↑PINK1, ↑Parkin, ↑LC3/TOMM20 interaction	mice	200 µmol/kg, ip	Liu et al. (2019)
		↓ROS, ↑SOD activity, ↓MDA, ↓Bax/Bcl-2, ↓Cyt-c			
Dendrobium nobile Lindl. alkaloids	35 and 350 ng/ml	↔PINK1, ↔Parkin, ↓p62, ↓LC3-II/LC3-I	PC12 cells	300 µM	Fu et al. (2021)
		↔Bcl-2, ↓Bax			
		↓ROS, ↓Apoptosis, ↔Mitochondrial function			
Corynoxine B	100 µM	↓p62, ↑LC3-II/LC3-I, ↓HMGB1, ↑HMGB/Beclin1 interaction, ↓Bcl-2/Beclin 1 interaction, ↓p-mTOR, ↓p-4EBP, ↓p-S6K1, ↔cell viability, ↓apoptosis	SH-SY5Y cells	50, 100, 200 µM	Yan et al.(2019)
Resveratrol	25 µM	↓LC3-II/LC3-I, ↑p62, ↔ mitochondrial function, ↔cell viability, ↔GSH/GSSG	PC12 cells	300 µM	Zhou et al. (2018)
Curcumin	300 mg/kg, ig	↑nuclear TFEB, ↑Beclin 1, ↑LC3-II/LC3-I, ↓p62, ↓p-mTOR, ↓mTOR, ↓α-Synuclein	rats (male)	15 mg/kg, ip	Lai et al. (2022)
**Synthetic compounds**					
N-acetylcysteine	1 mM	↓LC3-II/LC3-I, ↑p62, ↔ mitochondrial function, ↔cell viability, ↔GSH/GSSG	*In vitro*, PC12 cells	300 µM	Zhou et al. (2018)
LRRK2-IN-1	*In vitro*, 10 nM; *in vivo* (unk)	↓Beclin 1, ↓Atg5, ↓LRRK2, ↓IL-1β, ↓TNF-α	*In vitro* (BV-2 cells) and *in vivo* (mice, male)	*In vitro* (unk); *in vivo* (100 mg/kg, ip)	Chen et al. (2018)
1400 W	20 µM	↓p-parkin, ↓LC3B, ↓mitophagy, ↔mitochondrial damage, ↓apoptosis, nitrosative stress	*In vitro*, primary rat neurons	200 µM	Liu et al. (2022)
	5, 10, 20 µM (*in vitro*) 5-20 µmol/kg, sc (*in vivo*)	↑autophagolysosomes ↑LC3-II/LC3-I, ↑Beclin 1, ↓Bcl-2 /Beclin1 interaction, ↓apoptosis	*In vitro*, SH-SY5Y; *In vivo*, Mice (male & female)	*In vitro*, 200 µM; *in vivo*, 300 µmol/kg, ip	Ma et al. (2020)

ip, intraperitoneal; in, instranasal; po, per oral; sc, subcutaneous; ig, intra-gastric; ovx, ovariectomized; αSyn, α-synuclein; CAT, catalase; ER, estrogen receptor; ERK, extracellularly regulated kinase; GSH, glutathione; GSSG, glutathione disulfide; Glu, glutamate; Gln, glutamine; GS, glutamine synthase; GLAST, glutamate aspartate transporter; GLT-1, glutamate transporter 1; HMGB1, high mobility group box 1protein; IL-1β, interleukin 1β; iNOS, inducible nitric oxide synthase; LC3, microtubule-associated protein 1A/1B-light chain 3; LRRK2, leucine-rich repeat kinase 2; MAPK, mitogen activated protein kinase; MDA, malondialdehyde; mTOR, mammalian target of rapamycin; NO, nitric oxide; PARP, poly (ADP-ribose) polymerase; PAG, phosphate-activated glutaminase; PINK1, PTEN-induced kinase 1; p-ULK, phosphor-Unc-51, like autophagy activating kinase; p-4EBP, eukaryotic translation initiation factor 4E-binding protein; p-S6K1, ribosomal protein s6 kinase beta-1; PP2Ac, protein phosphatase 2A; PGE_2_, prostaglandin E2; ROS, reactive oxygen species; SOD, superoxide dismutase; TGF-α, transforming growth factor α; TOMM20, translocase of the outer mitochondrial membrane complex 20; TNF-α, tumor necrosis factor α; TFEB, transcription factor EB; unk, unknown.

### 4.1 Chelating agents

Given that chelating agents can remove heavy metals from the body (Andersen and Aaseth, 2016), PAS and EDTA have been shown to excrete Mn from the body and prevent acute Mn toxicity (Jiang et al., 2006; Zheng et al., 2009). PAS significantly decreased Mn levels in the body by 25–33% (Zheng et al., 2009). EDTA has also been extensively studied for the chelation therapy of toxic metals, including Mn, in their neurotoxicity (Discalzi et al., 2000). However, further studies are required to establish a safe treatment strategy as their therapeutic efficacy in decreasing morbidity and mortality is largely unestablished (Kosnett, 2010).

### 4.2 Antioxidants

As Mn-induced oxidative stress is one of the most studied mechanisms, numerous antioxidative therapeutics, including natural and synthetic, have been explored to mitigate Mn-induced oxidative stress. Many of those agents also exerted anti-inflammatory and antiapoptotic properties indirectly possibly due to secondary or downstream effects of their antioxidative functions to Mn-induced oxidative stress. Several antioxidants have been summarized, including polyphenols, natural peptides, and fatty acid derivatives against Mn-induced neurotoxicity (Table 1).

#### 4.2.1 Natural compounds

Studies have shown that resveratrol, a type of natural phenol, effectively attenuated Mn-induced toxicity by enhancing SOD, and CAT activities, resulting in reduction of Mn-elevated ROS levels and lipid peroxidation in mice and rats (Gawlik et al., 2017; Cong et al., 2021) as well as primary cortical neurons and PC12 cells (Sun et al., 2021). The molecular mechanisms involved in resveratrol’s protective effects against Mn-induced toxicity may involve multiple targets, including Nrf2-HO-1 and sirtuin 1 and 3 (Zhao et al., 2019; Sun et al., 2021). Polyphenol flavonoids are abundant in food sources, and several flavonoid polyphenols, including quercetin, silymarin, and curcumin, have shown to exhibit antioxidative properties against Mn-induced toxicity. Quercetin which has been shown to exert neuroprotective effects against various neurodegenerative disease models (for review (Islam et al., 2021), induced protective effects against Mn toxicity by attenuating Mn-induced impairment of SOD and CAT activities in the rat brain (Adedara et al., 2017; Gawlik et al., 2017). Quercetin also activated antioxidative Nrf2-HO-1 signaling to mitigate Mn-induced production of ROS, malondialdehyde (MDA), and protein carbonyls in SK-N-MC cells and rats (Bahar et al., 2017b). Silymarin from milk thistle (*Silybum marianum*) exerted antioxidative and neuroprotective effects against Mn toxicity by attenuating Mn-impaired SOD, GPx, and CAT activity, resulting in reduction of oxidative stress in the rat brain (Chtourou et al., 2010). Curcumin from turmeric exerted antioxidative effects by reducing Mn-increased MDA and ROS levels, while promoting HO-1 activity in BV-2 microglia (Park and Chun, 2017a). Curcumin also enhanced antioxidant effects by increasing activities of CAT, SOD1, and SOD2, and reversed Mn-induced epigenetic dysregulation by modulating H3K18 acetylation and H3K27 acetylation along with attenuation of Mn-induced oxidative stress and dopaminergic toxicity in the rat brain (Yang et al., 2022). Rutin, a polyphenolic flavonoid glycoside, mitigated Mn-induced toxicity in rats by attenuating Mn-dysregulated antioxidant SOD, CAT, and GPx activities (Nkpaa et al., 2019). Polyphenol flavonoids from *Euphorbia supina* weed have shown strong antioxidant properties and protective effects against Mn-induced neurotoxicity by attenuating Mn-induced SOD and CAT dysregulation in SK-N-MC cells and the rat brain (Bahar et al., 2017a). Anthocyanin from acai berries has been shown to exert antioxidative effects by attenuating Mn-dysregulated GSH/GSSG (glutathione disulfide) and lipid peroxidation partly by restoring Nrf2 levels and activity in rat primary astrocytes (da Silva Santos et al., 2014).

Several peptides and lipid compounds also showed antioxidant capabilities against Mn-induced toxicity in both *in vitro* and *in vivo* models. Carnosine (β-alanyl-l-histidine), a natural dipeptide commonly found in meat, exhibited its protective properties as a molecular chaperone and inducer of antioxidant systems and reduced Mn-induced lipid peroxidation in Mn-exposed mice (Ommati et al., 2020). Taurine found in animal tissues has shown to reduce Mn-induced oxidative stress and mitochondrial damage by reducing lipid peroxidation and enhancing GSH and total antioxidant capacity in mice (Ahmadi et al., 2018; Ommati et al., 2019). Macamides, long-chain fatty acid N-benzylamides from *Lepidium meyenii* or Maca, showed antioxidant properties (Almukadi et al., 2013) and exerted protection against Mn-induced neurotoxicity by increasing GSH levels, mitochondrial membrane potential, and PPAR-γ levels in U97 MG cells (Gugnani et al., 2018). Lipoic acid, which is synthesized in animals normally, acting as a cofactor for enzymes such as pyruvate dehydrogenase for the citric acid cycle, exerted protective effects against Mn toxicity by attenuating Mn-induced lipid peroxidation and dysregulated GPx levels in rats (Ibrahim et al., 2020). Boldo (*Peumus boldus*) leaf extracts which contain alkaloids, such as boldine, isoboldine, and N-methyllaurotetanine, displayed antioxidant activities, reducing lipid peroxidation and mortality in a *Drosophila* model of Mn-induced toxicity (Bianchini et al., 2016).

#### 4.2.2 Synthetic compounds

*N*-acetylcysteine (NAC), a precursor of GSH, effectively reduced Mn-induced oxidative damage and apoptosis in SH-SY5Y cells (Stephenson et al., 2013). NAC also attenuated Mn-dysregulated GR and GPx levels in SH-SY5Y cells (Maddirala et al., 2015) and increased SOD and CAT activities in the rat brain (Chopra et al., 2021).

Diphenyl diselenide, an organoselenium compound, increased antioxidant GPx and GSH activities (Orian and Toppo, 2014) and attenuated Mn-induced oxidative stress in Drosophila (Adedara et al., 2016). Another organoselenium, Ebselen, also attenuated Mn-induced dysregulation of antioxidant GSH in rats (Santos et al., 2012).

Vinpocetine, a synthetic derivative of the vinca alkaloid vincamine, attenuated Mn-induced oxidative stress and apoptosis in rats (Nadeem et al., 2018) and NE-4C cells (Bora et al., 2016). D-ribose-L-cysteine, a cysteine analog exhibiting antioxidant properties, attenuated Mn-induced oxidative stress and toxicity by enhancing CAT and SOD activity in rats (Akingbade et al., 2022).

Edaravone (Radicava), an FDA-approved drug for treating stroke and amyotrophic lateral sclerosis (ALS) (Bhandari et al., 2018), has shown its therapeutic efficacy against Mn-induced toxicity by reducing Mn-induced lipid peroxidation, protein degradation, and mitochondrial dysfunction in the rat brain as well as primary astrocytes (Evren et al., 2015; Apaydin et al., 2016).

In addition to its chelating properties on Mn, PAS exerted protective effects against Mn-induced neurodegeneration by enhancing antioxidant, anti-inflammatory, anti-excitotoxicity, and antiapoptotic effects in various *in vitro* and *in vivo* experimental models (Zheng et al., 2009; Ou et al., 2011; Santos et al., 2012; Li et al., 2017; Peng et al., 2020; Deng et al., 2021). Organotellurium compounds have shown antioxidant activities in the brain. For example, organotellurium diethyl-2-phenyl-2-telluropheny vinyl phosphonate (DPTVP) exerted neuroprotection against Mn-induced neurotoxicity and behavioral deficits in rats, attributing to its antioxidant activity (Avila et al., 2010) and attenuated Mn-induced reduction in survival in *C. elegans* (Avila et al., 2012). Dithiolethiones, lipophilic organosulfur compounds that activate Nrf2, upregulated various phase II antioxidant enzymes, and its disubstituted dithiolethione (ACDT) exerted protection against Mn toxicity by increasing GSH, NAD(P)H dehydrogenase in SH-SY5Y cells (Kulkarni et al., 2021).

Trolox, a water-soluble vitamin E analog, attenuated Mn-induced ROS, F2-isoprostanes, and caspase activity in the rat striatum and rat primary neurons (Milatovic et al., 2011; Cordova et al., 2013). Probucol, an anti-hyperlipidemic drug, showed powerful antioxidative effects, attenuating Mn-induced ROS production, mitochondrial dysfunction, and cell death in SH-SY5Y neuronal and C6 glial cells (da Silva et al., 2022). Minocycline, a tetracycline antibiotic, exerted antioxidative effects against Mn-induced oxidative stress by decreasing lipid peroxidation and increasing antioxidant enzymes such as SOD in Drosophila (Bonilla et al., 2012; Mora et al., 2014). Melatonin, an endogenous hormone produced by the pineal gland, has been shown to attenuate Mn-induced motor dysfunction and neuronal loss in mice by inhibiting Mn-induced oxidative stress (Deng et al., 2015). Other synthetic antioxidants have also shown efficacy in treating Mn-induced neurotoxicity in various experimental models, but it has yet to be established in clinical trials whether they are efficacious in humans as well.

### 4.3 Anti-inflammatory compounds

Accumulating evidence reveals that Mn toxicity promotes neuroinflammation by increasing proinflammatory cytokines such as TNF-α, ILs, and inflammatory mediator COX-2. The non-steroidal anti-inflammatory drug (NSAID) indomethacin has been shown to alleviate Mn-induced neuroinflammation, oxidative stress, and energy dysregulation in rat primary cortical neurons and mice (Milatovic et al., 2011). The antibiotic, minocycline, inhibited Mn-induced macrophage-mediated inflammation with concomitant attenuation of Mn-reduced striatal TH of the rat brain (Ponzoni, 2012).

The calpain inhibitor, calpastatin, has been shown to attenuate Mn-induced toxicity by reducing proinflammatory markers, including IL-1β, TNF-α, NF-κB in the rat hippocampus and striatum, preventing Mn-induced motor deficit and microglial Iba1 expression and dopamine dysregulation in rat striatum (Ivleva et al., 2022).

Melatonin, which has been shown to afford neuroprotection *via* its anti-inflammatory and antioxidant properties in PD and AD models (Asefy et al., 2021) also attenuated Mn-induced increase in proinflammatory cytokines, such as TNF-α and IL-1β and IκB-α in BV-2 cells (Park and Chun, 2017b). Anti-inflammatory agents showing efficacy against Mn-induced neurotoxicity are listed in Table 2.

### 4.4 Glutamate excitotoxicity modulators

Mn can lead to accumulation of glutamate in the synapse and excitotoxicity (Lee et al., 2009b) by impairing astrocytic glutamate transporters and overstimulating the NMDA receptor (NMDAR). Several compounds have been shown to increase expression of the glutamate transporters EAAT1 (GLAST) and EAAT2 (GLT-1) (Pajarillo et al., 2018), and some agents modulated NMDAR and α-amino-3-hydroxy-5-methyl-4-isoxazolepropionic acid (AMPA) receptors and attenuated Mn-induced neurotoxicity. The current findings on pharmacological agents that modulate glutamate transporters and receptors to protect against Mn toxicity were reviewed in the following sections and summarized in Table 2.

#### 4.4.1 Estrogen and SERMs

Estrogens (mainly 17β-estradiol, E2) and selective estrogen receptor modulators (SERMs) have been shown to exert neuroprotective effects in various experimental models of neurodegenerative disorders and Mn’s neurotoxicity. E2 and tamoxifen (TX) attenuated Mn-reduced glutamate uptake and GLT-1/GLAST expression by upregulation of GLT-1 and GLAST at the transcriptional level in rat primary astrocytes (Lee et al., 2009a). E2 and TX increased GLT-1 by increasing transforming growth factor α in astrocytes (Lee et al., 2012). E2 and TX have also been shown to attenuate Mn-induced reduction of GLT-1/GLAST, along with locomotor activity and dopaminergic neuronal injury in mice (Pajarillo et al., 2018).

Another SERM, raloxifene (RX), attenuated Mn-reduced GLT-1 expression and glutamate uptake by upregulating GLT-1 protein expression in rat primary astrocytes (Karki et al., 2014b). Several signaling pathways, such as MAPK/ERK, PI3K/Akt and EGFR, and TFs, such as CREB and NF κB were involved in E2/TX/RX-increased GLT-1 expression. Moreover, the estrogen receptors, ER-α, ER-β, and GPR30, mediated GLT-1 transcription *via* both genomic and non-genomic mechanisms (Karki et al., 2014b). These findings suggest that various pathways are involved in E2-and SERMs-induced GLT-1/GLAST expression and neuroprotection against Mn’s excitotoxic neuronal injury (Pajarillo et al., 2019).

#### 4.4.2 Epigenetic modulators

Given that HDAC suppresses gene transcription (Mai et al., 2009) and Mn activates HDACs, inhibiting HDACs could be potential therapeutic targets for treating Mn toxicity. Studies have shown that HDACi such as valproic acid (VPA) and sodium butyrate (NaB) increased GLAST/GLT-1 expression and attenuated Mn-reduced GLAST/GLT-1 in both *in vitro* and *in vivo* models (Johnson et al., 2018b). The antiepileptic VPA has been shown to exert neuroprotective effects in Mn toxicity by attenuating Mn-induced decrease in TH mRNA and protein levels as well as motor deficits in astrocyte cultures and mouse brain tissue (Johnson et al., 2018a). NaB also attenuated Mn-reduced GLT-1/GLAST in cortical and cerebellar regions of mice, along with motor function deficits. These findings suggest that HDACi could exert neuroprotection against Mn toxicity, at least in part by upregulation of astrocytic glutamate transporters GLT-1/GLAST. Moreover, modulating DNA methylation and miRNA could also be potential targets to treat Mn toxicity as a demethylation reagent 5-Aza-dC attenuated Mn-induced inflammation in microglia (Liu et al., 2022b), and inhibition of Mn-activated miRNAs attenuated Mn’s relevant target genes in SH-SY5Y cells (He et al., 2017; Tarale et al., 2018).

#### 4.4.3 Arundic acid

Arundic acid [(R)-(-)-2-propyloctanoic acid (ONO-2506)] was first discovered as an inhibitor of S100β, a calcium-binding protein produced primarily in astrocytes, ameliorating ischemic brain damage in rats (Tateishi et al., 2002), as well as MPTP-induced PD mice by modulating astrocytic activation (Kato et al., 2004; Himeda et al., 2006). Arundic acid exerted protective effects against Mn toxicity by inhibiting Mn-induced downregulation of GLAST/EAAT1 and GLT-1/EAAT2 mRNA and proteins levels in human H4 astrocytes and rat primary astrocytes (Karki et al., 2018). This effect was mediated by the activation of the Akt and ERK pathways as well as the inhibition of Mn-activated YY1 that is a repressor of EAAT1/2 (Karki et al., 2018).

#### 4.4.4 Fluoxetine and riluzole

Fluoxetine, which is clinically used to treat depression due to serotonin (5-HT) uptake inhibition, has increased GLT-1 expression in the rat brain (Chen et al., 2014). Riluzole, a voltage-dependent Na channel blocker and is used to treat ALS, has been shown to inhibit glutamate release from neuronal synapses (Irifune et al., 2007) and increase glutamate reuptake by astrocytes (Fumagalli et al., 2008). Both fluoxetine and riluzole attenuated the Mn-induced increase in ephrin A3 and decrease in GLT-1 and GLAST mRNA/protein levels in primary striatal murine astrocytes (Deng et al., 2009; Qi et al., 2020).

#### 4.4.5 Diclozipine (MK-801) and dextromethorphan

MK-801 and dextromethorphan (DM) are non-competitive NMDAR antagonists. MK-801 inhibited the NMDAR to prevent Ca^2+^ overload and ROS production (Thomas and Kuhn, 2005), and DM protected the rat brain cortex against brain trauma injury by anti-excitatory and anti-inflammatory mechanisms (Pu et al., 2015). Importantly, both MK-801 and DM attenuated Mn-induced neurotoxicity by inhibiting the NMDAR overstimulation in rats (Xu et al., 2010b). MK-801 attenuated Mn-induced NMDA excitotoxic lesion, along with ATP reduction and decreased dopamine and GABA levels in the rat striatum, corroborating that Mn may produce neuronal degeneration by an indirect excitotoxic process secondary to its ability to impair oxidative energy metabolism (Brouillet et al., 1993).

### 4.5 Autophagy and mitophagy modulators

#### 4.5.1 Natural compounds

Trehalose is a disaccharide comprised of two glucose molecules which is synthesized in bacteria, fungi, plants, and invertebrate animals. Trehalose has been shown to afford protective effects against Mn-induced neurotoxicity in the murine brain by increasing autophagy activation along with attenuation of Mn-induced oxidative stress and α-Syn oligomerization (Jing et al., 2020). Trehalose mitigated Mn-induced PINK1/parkin-dependent mitophagy dysregulation by attenuating Mn-increased cleaved-poly (ADP-ribose) polymerase (PARP) and mitochondria-containing autophagic lysosomes, resulting in inhibition of Mn-induced mitochondrial damage and apoptosis in mice (Liu et al., 2019).

Alkaloids from Dendrobium nobile have been shown to attenuate Mn-impaired autophagy and mitophagy proteins, including LC3-II, p62, PINK1, and parkin, and inhibition of Mn-induced oxidative stress, apoptosis, and mitochondrial dysfunction in PC12 cells (Fu et al., 2021). Corynoxine B, a natural oxindole alkaloid from Mitragyna speciose and Uncaria macrophylla, exerting protective effects in experimental models of AD and PD (Zeng et al., 2019; Chen et al., 2021; Zhu et al., 2022), attenuated Mn-impaired mTOR signaling and autophagy in SH-SY5Y cells (Yan et al., 2019). Studies have also shown that resveratrol and curcumin led to protection against Mn-induced neurotoxicity by promoting autophagy in both PC12 cells and rats (Zhou et al., 2018; Lai et al., 2022).

#### 4.5.2 Synthetic compounds

Elevated LRRK2 kinase activity is closely related to inflammatory signaling, and Mn activated LRRK2, leading to an increase in autophagy proteins such as Beclin 1 and TNF-α levels in microglia (Chen et al., 2018; Kim et al., 2019). LRRK2-IN-1, an inhibitor of LRRK2, attenuated Mn-induced autophagy by inhibiting Mn-increased Beclin 1 and Atg5 in BV-2 microglia and mice, indicating that LRRK2 is involved in Mn-induced autophagy dysregulation and inflammation (Chen et al., 2018).

1400 W is a selective inhibitor of iNOS, exerting anti-inflammatory and antioxidative properties against various neurodegenerative disease models. 1400 W alleviated Mn-induced autophagic impairment and neuronal injury by increasing autophagic vacuoles and protein levels of Beclin 1, LC3-II, while decreasing p62 levels along with inhibiting Mn-induced nitrosylation of JNK, IKKβ, and Bcl-2 in mice and SH-SY5Y cells (Ma et al., 2020). NAC mitigated Mn-induced autophagy dysfunction likely *via* its anti-oxidative properties (Zhou et al., 2018).

## 5 Conclusion

The underpinning of mechanisms of Mn-induced neurotoxicity provides critical information for developing neurotherapeutics and treatment strategies. Accordingly, delineating the transcriptional and intracellular pathways involved in the regulation of oxidative stress, autophagy, mitophagy, inflammation, and excitotoxicity is critical to further our understanding of Mn-induced neuropathogenesis. To date, no single agent has shown to be a promising therapeutic to treat Mn toxicity, warranting further investigation of the underlying mechanism and, thus, developing therapeutics using precise molecular targets to treat or prevent Mn’s neurotoxicity.

### 5.1 Future direction

Targeting specific molecules modulating various mechanisms induced by Mn toxicity, including autophagy, mitophagy, excitotoxicity, as well as Mn’s systemic removal are exciting avenues for exploration, expanding our comprehensive knowledge in developing newer, efficacious, specific gene and drug therapies for manganism and other related neurological disorders. Moreover, since Mn targets multiple genes and proteins, combination therapy strategies comprising of several relevant targets could be more beneficial for optimal treatment against Mn neurotoxicity.

## Glossary

**E2**: 17β-estradiol
**AD**: Alzheimer’s disease
**ARE**: antioxidant response element
**COR**: carboxy-terminal of ras
**CAT**: Catalase
**DM**: dextromethorphan
**DPTVP**: diethyl-2-phenyl-2-telluropheny vinyl phosphonate
**Drp-1**: dynamin-related protein 1
**EDTA**: ethylenediaminetetraacetic acid
**EAAT**: excitatory amino acid transporter
**GLT-1**: glutamate transporter 1
**GLAST**: glutamate aspartate transporter
**GSH**: glutathione
**GPx**: glutathione peroxidase
**GSSG**: glutathione disulfide
**HO-1**: heme oxygenase 1
**HDACi**: histone deacetylase inhibitors
**HRE**: hypoxia response elements
**HIF1α**: hypoxia-inducible factor 1α
**IL**: interleukin
**KHSRP**: K-homology splicing regulator protein
LRRK: leucine rich repeat kinase
Mn: manganese
Mfn2: mitofusin 2
NOS2: nitric oxide synthase 2
**NMDA**: *N*-methyl-D-aspartate
**NMDAR**: NMDA receptor
**NSAID**: non-steroidal anti-inflammatory drug
**NF-κB**: nuclear factor kappa-light-chain-enhancer of activated B cells
**Opa-1**: optic atrophy type 1
**PAS**: para-aminosalicylic acid
**PD**: Parkinson’s disease
**PPAR-γ**: peroxisome proliferator-activated receptor gamma
**PINK1**: phosphatase and tensin homolog induced kinase 1
**RX**: raloxifene
**REST**: RE1-silencing transcription factor
**ROS**: reactive oxygen species
**RE1**: repressor element 1
**SERMs**: selective estrogen receptor modulators
**NaB**: sodium butyrate
**SOD**: superoxide dismutase
**TX**: tamoxifen
**TNF-α**: tumour necrosis factor α
**TFs**: transcription factors
**TFEB**: transcription factor EB
**TH**: tyrosine hydroxylase
**VPA**: valproic acid
**Wip 1**: wild type p53-induced phosphatase 1
**YY1**: Yin Yang 1
**αSyn**: α-Synuclein
